# Supplementary material for: Causal Relationship between Aging and Anorexia Nervosa: A White-Matter-Microstructure-Mediated Mendelian Randomization Analysis
Source: Biomedicines. 2024 Aug 16;12(8):1874. doi: 10.3390/biomedicines12081874 (PMC11351342; doi:10.3390/biomedicines12081874)
Supplement: Supplementary file 1 [file biomedicines-12-01874-s001.zip › Supplementary Figure/Figure S8/Leaveoneout plot of MR analysis for TL to ubm-b-1985.pdf]

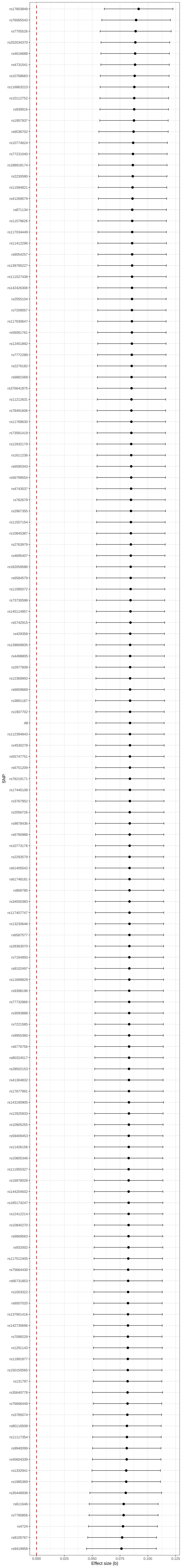
rs17803849
rs76065543
rs7705526
rs202034370
rs4616688
rs4731541
rs10768683
rs116863223
rs10112752
rs939916
rs1957937
rs6536702
rs10774624
rs77231040
rs188918174
rs2230590
rs11584821
rs41269079
rs871134
rs11579626
rs117034449
rs11412296
rs6054257
rs139795227
rs111527438
rs142426306
rs2555104
rs7209057
rs117630647
rs56061761
rs12451892
rs7772289
rs2276182
rs6881568
rs376641875
rs11212631
rs78491606
rs11769630
rs73581419
rs12932179
rs1611236
rs6590343
rs56799554
rs4743037
rs762679
rs2967355
rs11557154
rs10845387
rs2763979
rs4695407
rs182059586
rs6584579
rs11085072
rs73730598
rs145114957
rs5742915
rs429358
rs139669835
rs4498805
rs2977608
rs12369950
rs6659669
rs3891167
rs1907702
All
rs112394943
rs4530278
rs55747751
rs6751209
rs76219171
rs17445108
rs3767952
rs2056726
rs9878436
rs6790988
rs10773176
rs2293579
rs61405042
rs61748181
rs869785
rs34550383
rs117407747
rs13230646
rs6587577
rs28363070
rs7164950
rs8102497
rs11699829
rs9398196
rs77732866
rs3093888
rs7221585
rs9955360
rs6776756
rs80324517
rs28502153
rs41304832
rs17677991
rs143190905
rs12925933
rs10905255
rs59409453
rs11426156
rs10805346
rs111950327
rs16978028
rs144204502
rs185174247
rs12412214
rs10840270
rs6669563
rs932002
rs117512405
rs75664430
rs66731853
rs1003322
rs6007020
rs137901416
rs142730696
rs7099229
rs1291143
rs11991877
rs150150565
rs131797
rs35640778
rs76666449
rs3785074
rs80116508
rs11117354
rs9940099
rs45604339
rs1332941
rs1985369
rs35446936
rs611646
rs7790856
rs4724
rs8105767
rs9419958
SNP
0.000
0.025
0.050
0.075
0.100
0.125
Effect size (b)
